# Sox2 modulates motility and enhances progression of colorectal cancer via the Rho-ROCK signaling pathway

**DOI:** 10.18632/oncotarget.21709

**Published:** 2017-10-10

**Authors:** Junheng Zheng, Lixiao Xu, Yubin Pan, Shuyi Yu, Hongbo Wang, Derek Kennedy, Yan Zhang

**Affiliations:** ^1^ Key Laboratory of Gene Engineering of the Ministry of Education, State Key Laboratory of Biocontrol, School of Life Sciences, Sun Yat-sen University, Guangzhou, China; ^2^ Sun Yat-sen University-Griffith University Joint Laboratory for Drug Discovery, Guangzhou, China; ^3^ Sun Yat-sen University Cancer Center, Guangzhou, China; ^4^ Griffith Institute for Drug Discovery, Griffith University, Brisbane, Queensland, Australia

**Keywords:** Sox2, colorectal cancer, cancer stem cell, motility, ROCK

## Abstract

Sox2 (Sry-box2) is essential for a variety of stem cells and is also expressed in colorectal cancer (CRC). However, the underlying mechanism by which Sox2 enhances CRC progression remains unclear. In the present study, we show that elevated Sox2 expression is significantly correlated with poor clinical prognosis. CRC is phenotypically heterogeneous, and harbors several subtypes of cancer cells. Elevated Sox2 expression was always detected in rounded-shape cells, which co-located to poorly differentiated regions, the invasive frontier and metastatic lesions. Knockdown of Sox2 in CRC cells not only decreased the number of round-shaped cells, but also suppressed cell migration, invasion as well as attenuated colony forming capacity and tumorigenicity. By contrast, overexpression of Sox2 in CRC cells was associated with up-regulation of multidrug resistance genes and accelerated CRC progression. Moreover, Sox2 conferred activation of Rho-ROCK signaling, whereas inhibition of ROCK signaling decreased cell migration, invasion, colony formation and self-renewal of CRC. Our results reveal that CRC is phenotypically and functionally heterogeneous. Elevated Sox2 expression activates the Rho-ROCK pathway, which in turn changes cell morphology and promotes cell migration and progression.

## INTRODUCTION

Colorectal cancer (CRC) is a major cause of cancer-related deaths worldwide [1]. Approximately 50% of CRC patients can be treated by surgery and multimodal treatment before disease progression. However, 40-50% of patients have metastatic disease which requires more aggressive treatment. Reduced mortality has been attributed to earlier detection and improvements in therapy. However, early diagnosis of individuals at high risk of metastasis is hampered by the lack of understanding of the underlying mechanisms.

Cancer invasion and metastasis are tightly associated with the acquisition of a migratory phenotype. Two different modes of cell movement are identified in individual tumor cells: a mesenchymal mode characterized by an elongated morphology that requires extracellular proteolysis localized at cellular protrusions and an amoeboid mode, in which movement is independent of proteases and typically these cells have a rounded morphology. Cell movement is regulated by members of the Rho family. Rho signals to ROCK, promoting the formation of actin stress fibers and generation of the actomyosin contractile force, whereas Rac1 and Cdc42 drive motility by promoting lamellipodia formation [2, 3]. The amoeboid mode involves signaling through ROCK, whereas the mesenchymal mode requires extracellular proteolysis for Rac-dependent actin protrusions to be pushed through channels in the extracellular matrix [4].

The transcription factor Sox2 plays an important role in various phases of embryonic development and affects cell fate and differentiation [5]. Recently, Sox2 has been studied in several types of human solid tumors [6–8]. In CRC, Sox2 is associated with stemness, growth and metastasis [9–11]. However, further studies are required to determine the molecular pathways associated with these biological functions.

The presence of cancer stem cells (CSCs) in CRC triggers tumor initiation, metastasis, relapse and chemoresistance [12, 13]. Our previous work showed that the CRC cell line, SW620, contained a sub-population with CSCs characteristics. The stem-like cells in SW620 were able to self-renew, resisted chemotherapy, initiated tumors efficiently in nude mice, and formed Sox2-enriched spheres *in vitro* [14].

In this study, we showed that a high expression level of Sox2 was detected in CRC cells with aggressive capacities and stemness. Compound Y27632, which blocks ROCK signaling, was used to demonstrate that Sox2 expression is associated with Rho-Rock- dependent cell morphology and CRC progression.

## RESULTS

### Expression of Sox2 in CRC tissues and cell lines

**Figure 1:**
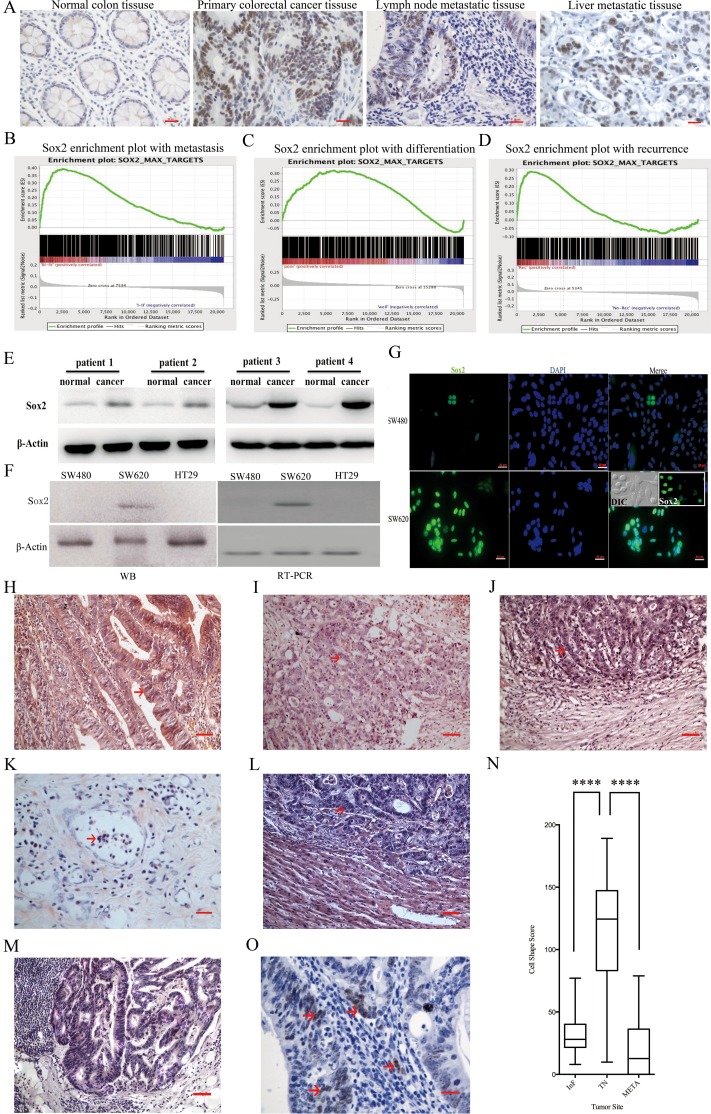
Sox2 expression in clinical tumor tissues and cell lines **(A)** Immunohistochemical staining of Sox2 in paraffin-embedded human colorectal tissues. Scale bar: 20 μm. **(B-D)** GSEA assay. **(E)** WB analysis of Sox2 in CRC tissues and normal counterparts. **(F)** Detection of Sox2 in CRC cell lines by WB (left) and RT-PCR (right). **(G)** Immunofluorescent staining of Sox2 in SW480 and SW620. Rounded cells expressed Sox2 at high levels (inset). Nuclei were stained by DAPI, matched laser power was used when photographing the images. Scale bar: 20 μm. **(H)** Luminal structure located in a tumor nest. Scale bar: 20 μm. **(I)** Poorly differentiated lesion. Scale bar: 20 μm. **(J)** Representative image of an InF. Scale bar: 20 μm. **(K)** Representative image of a vessel embolus. Scale bar: 10 μm. **(L)** Representative image of a liver metastatic lesion. Scale bar: 20 μm. **(M)** Cancer cells in different stages of differentiation were observed in lymph node metastatic lesions. Scale bar: 20 μm. **(N)** The evaluation of CSS scores. **(O)** Higher expression levels of Sox2 were detected in rounded cells. Arrows indicate Sox2 expression. Scale bar: 10 μm. Student's t test was used for statistic analysis, ^****^ p < 0.001.

136 specimens, including CRC tissues, paratumoral tissues, lymph node metastatic tissues and distant organ metastatic tissues were evaluated for the Sox2 expression by immunohistochemistry. As shown in Figure 1A, significantly higher expression of Sox2 was observed in the primary CRC tissues and metastatic tumor tissues as compared to paratumoral tissues with 80.85% of the analyzed primary tumor tissues exhibiting elevated Sox2 expression. Additionally, 76.47% of the lymph node metastatic tissues and 70% of the distant organ metastatic tissues showed a high level of Sox2 expression (Table 1).

**Table 1:** Sox2 expression in colorectal cancer tissues

		Sox2 Expression		Density Mean	
Variables	No.	Negative (%)	Positive (%)	Mean±SD (×10^-4^)	*p*
**Location**					
**Normal**	45	28 (62.22)	17 (37.78)	8.63±0.88	
**Cancer**	47	9 (19.15)	38 (80.85)	35.59±4.24	<0.001
**Lymph node metastic**	34	8 (13.53)	26 (76.47)	27.03±4.11	0.003
**Other organs metastic**	10	3 (30.00)	7 (70.00)	19.35±3.98	0.0193
**Grade**					
**Duke's A**	5	0 (0)	5 (100.00)	15.31±1.93	0.002
**Duke's B**	4	0 (0)	4 (100.00)	51.88±12.34	
**Duke's C**	23	5 (21.74)	18 (78.26)	0.0024.07±2.88	0.003
**Duke's D**	15	4 (26.67)	11 (73.33)	18.20±2.18	<0.001
**Five year survival data**					0.039
**Dead**	26	5 (16.00)	21 (84.00)	53.36±9.94	
**Live**	15	1 (6.67)	14 (93.33)	30.44±4.63	

Clinical characteristics and Sox2 expression in 136 specimens of human colorectal tissues are presented. With respect to location: *p* stands for the Significant difference of staining density between normal tissues and other tissues. With respect to grade: *p* stands for the significant difference of staining density between Grade B and the other identified Grades.

Tumor samples used in this study were classified according to Duke's grade, and all tumors of grade A or B expressed Sox2, with especially high expression in grade B. Tumors of grade C or D also demonstrated a high frequency of Sox2 expression, suggesting that Sox2 could be detected in the early stages of CRC. Furthermore, the Sox2 expression level was higher in metastatic than in non-metastatic tumors. Additionally, elevated Sox2 expression was correlated to the reduced 5-year survival rate. Using data from recently published studies, a Sox2-target gene set was selected and used on a representative pooled cohort of 232 patients with primary CRC (GSE17536). GSEA revealed that the expression of a Sox2-targeted gene set was strongly associated with metastasis in CRC (Figure 1B). Furthermore, the Sox2-targeted gene set was enriched in poorly differentiated tumors (Figure 1C) and recurrent cases (Figure 1D).

WB analysis showed that the Sox2 expression levels were significantly higher in cancer tissues than that in the normal counterparts (Figure 1E). Sox2 expression analysis in CRC-derived cell revealed elevated expression levels in SW620 cells when compared with HT29 and SW480 cells (Figure 1F and 1G). SW480 and SW620 cells were established from the same patient. SW480 cells were obtained from the primary lesion, while SW620 cells were derived from a lymph node metastatic lesion [15]. There are two types of cell morphology in the SW620 whole cell population; the rounded-shape and elongated-shape. Compared to elongated cells, the rounded cells showed strong Sox2 expression (Figure 1G and inset).

To interrogate the relationship between Sox2 expression and cell morphology in clinical CRC specimens, the CRC cell shape scores (CSS) were evaluated in 39 randomly selected CRC specimens. Several types of cancer cell morphology were identified, which range from rounded to elongated cell shape. Most luminal structures were composed of elongated cells (Figure 1H), while, the rounded cells were always found in solid lesions with poor differentiation (Figure 1I), the invasive frontier (InF, Figure 1J), vessel embolus (Figure 1K) and liver metastatic lesions (Figure 1L). In lymph node metastatic lesions and a sub-population of liver metastatic lesions, elongated tumor cells formed luminal structures and rounded cells constituted solid lesions resembling primary tumors (Figure 1M). The CSS of the InF or metastatic lesions (META) was significantly lower than that of tumor nests (TN, Figure 1N). Most of the well differentiated tumor cells in TN presented relative low Sox2 expression and showed an elongated shape. Moreover, the cells in the InF or META, which are more round and poorly differentiated, showed very strong Sox2 expression (Figure 1O).

### Sox2 is associated with cell migration, invasion, colony formation and tumorigenesis

**Figure 2:**
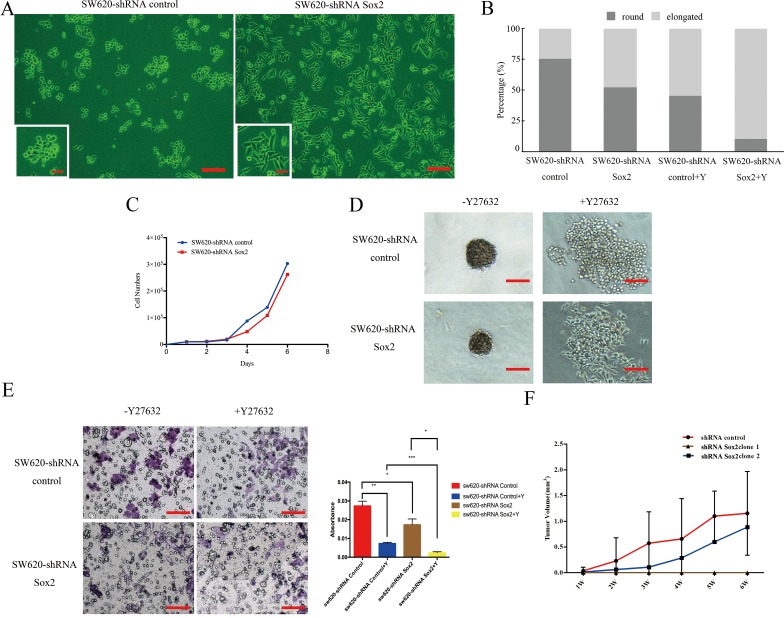
Knockdown of Sox2 inhibited colony formation, tumor cell migration, invasion and tumorigenesis *in vitro* and *in vivo* **(A)** Phase contrast of SW620-shRNA control cells and SW620-shRNA Sox2 cells. Scale bar: 100 μm. The magnification of the inserted images are ×200. **(B)** Quantification of the number of different shapes of cells with knockdown expression of Sox2 compared to the controls. Compound Y27632 (Y) was used to block Rho-ROCK signaling. **(C)** Growth assays of SW620-shRNA control cells and SW620-shRNA Sox2 cells. The cells (1×10^4^/ml) were seeded onto 24-well plates and cultured in DF12 containing 1% FBS. The cell numbers were counted every day as indicated. **(D)** Soft agar colony formation assays. Representative plates for each cell group are shown. **(E)** Invasion assay. The bar graph (right panel) presents the absorbance of crystal violet representing the number of invading cells. All of the experiments mentioned above were performed in triplicate. **(F)** Tumor-forming ability of SW620-shRNA control cells and SW620-shRNA Sox2 cells. Scale bar: 1 cm. Student's t test was used for statistic analysis, ^*^p< 0.05; ^**^ p < 0.01; ^***^ p < 0.005. Y represents Y27632.

Sox2 silencing significantly increased the number of elongated cells, and slightly inhibited cell proliferation (Figure 2A, 2B and 2C). SW620-shRNA Sox2 cells required longer incubation times to generate colonies of equivalent size to those generated by SW620-shRNA control cells (Figure 2D). In addition, knockdown of Sox2 reduced the capacity for cell invasion (Figure 2E). Furthermore, treatment with compound Y27632 further reduced the number of rounded cells (Figure 2B and Supplementary Figure 1), disrupted colony formation (Figure 2D) and reduced cell invasion (Figure 2E).

In order to study the role of Sox2 in tumorigenesis and tumorigenic progression, 1×10^6^ cells of each group were injected respectively into Balb/c nude mice subcutaneously. Compared with the tumors derived from SW620-shRNA control cells, two of the SW620-shRNA Sox2-derived tumors had smaller masses. Furthermore, cells within one of the SW620-shRNA Sox2-derived masses had lost its tumorigenic capacity. Notably, 1 in 8 animals injected with SW620-shRNA Sox2 clones developed tumors 4 weeks post-injection, whereas 100% of the SW620-shRNA control group formed tumors (Figure 2F). We also found that Sox2 expression levels in the SW620-shRNA Sox2-derived tumors were much lower than that in the control group, however, there was subtle difference of Sox2 expression level among SW620-shRNA Sox2-derived tumors (Supplementary Figure 2). We speculate here that different expression levels of Sox2 resulted in different tumorigenic capacity and tumor growth rate.

**Figure 3:**
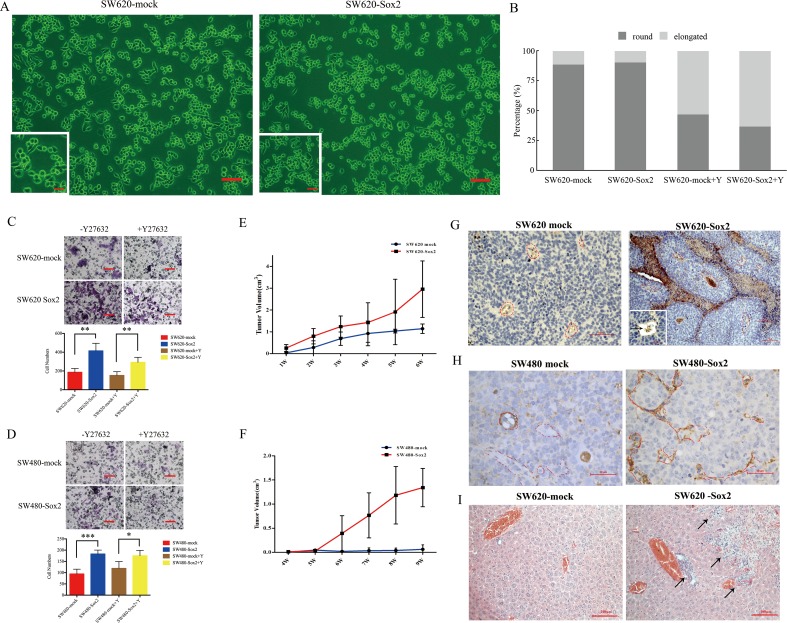
Overexpression of Sox2 promoted cell motility and tumorigenesis **(A)** Phase contrast of SW620-mock cells and SW620-Sox2 cells. Scale bar: 100 μm. The magnification of the inserted images are ×200. **(B)** Quantification of rounded cells and elongated cells. **(C)** Invasion assays for SW620-mock cells and SW620-Sox2 cells. **(D)** Invasion assays for SW480-mock cells and SW480-Sox2 cells. **(E)** Tumorigenicity of SW620-mock cells and SW620-Sox2 cells. **(F)** Tumorigenicity of SW480-mock cells and SW480-Sox2 cells. **(G)** CD31 immunostaining in the tumors derived from SW620-mock cells or SW620-Sox2 cells. Areas marked by red dashes represent tumor vessels. Arrows indicate tumor emboli (inset) within CD31-positive vessels or vessel-like structures. Scale bar: 50 μm. **(H)** CD31 immunostaining in the tumors derived from SW480-mock cells or SW480-Sox2 cells. Areas marked by red dashes represent tumor vessels. **(I)** H&E staining of the livers in SW620-mock group or in SW620-Sox2 group. Arrows indicate metastatic lesion in the liver, scale bar: 50 μm. Student's t test was used for statistic analysis, ^*^p< 0.05; ^**^ p < 0.01; ^***^ p < 0.005. Y represents Y27632.

To determine whether Sox2 is important in CRC etiology, SW620 cells and SW480 cells were stably transfected with Sox2. SW620 cells transfected with Sox2 (SW620-Sox2) showed slightly increased numbers of rounded cells (Figure 3A and 3B; Supplementary Figure 1). Increased expression of Sox2 promoted CRC cell proliferation and colony forming capacity (Supplementary Figure 3). In addition, Sox2-overexpressing cells showed increased invasive capacity (Figure 3C and 3D), suggesting that Sox2 affects the motility in both SW620 and SW480 cells. Y27632 reduced the number of rounded cells regardless of Sox2 overexpression (Figure 3B) and repressed colony formation capacity in SW620-mock cells but not in SW620-Sox2 cells (Supplementary Figure 3). Y27632 was not able to ameliorate the effects of Sox2 overexpression in invasion assays (Figure 3C and 3D).

Although both SW620-mock and SW620-Sox2 cells displayed a tumor formation rate of 75%, SW620-Sox2 cells presented increased growth rate and a larger tumor volume (2.9 ± 1.3 cm^3^) compared with SW620-mock cells (1.1 ± 0.2 cm^3^, Figure 3E) within the experimental period. In the SW480-mock group, 2 of 4 mice (50%) showed slow tumor growth after 9 weeks, while the SW480-Sox2 group displayed a tumor formation rate of 75% (n=4) and a final tumor volume of 1.3 ± 0.4 cm^3^ (Figure 3F). Pathological analysis showed that the xenografted tumors derived from the SW620-Sox2 or SW480-Sox2 cells exhibited proliferating CD31-positive capillaries or vessel-like structures. Furthermore, tumor emboli were frequently found in capillaries or vessel-like structures (Figure 3G and 3H). Notably, liver metastasis was found in the SW620-Sox2 group (Figure 3I). The results indicated that Sox2 might promote tumor cell migration and invasion into blood vessels, and eventually facilitate metastasis to the liver.

### Sox2 is required for spherogenicity and chemoresistance

**Figure 4:**
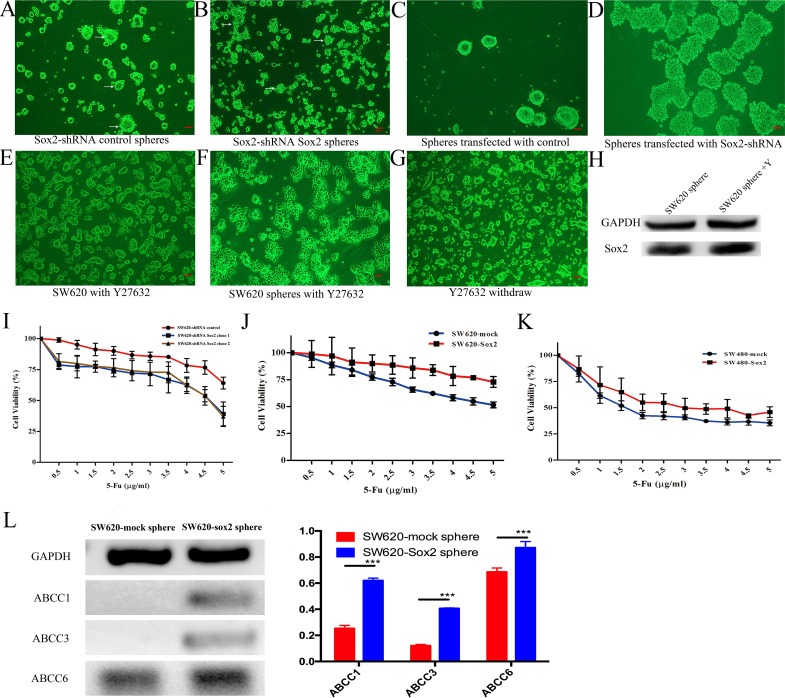
Sox2-Rho-ROCK signaling is required for CRC stem-like cells **(A)** Sphere forming cultures for SW620-shRNA control cells. Arrows indicate solid spheres. **(B)** Sphere forming cultures for SW620-shRNA Sox2 cells. Arrows indicate cell aggregates. **(C)** Morphology of stem-like spheres transfected with shRNA control. **(D)** Knockdown of Sox2 in stem-like spheres. **(E)** SW620 cells were treated with Y27632 for 2 days, and then cultured in stem cell medium. **(F)** SW620 spheres were treated with Y27632 for 1 day. **(G)** SW620-Sox2 cells formed spheres again after Y27632 removal. **(H)** WB analysis of Sox2 expression in SW620 spheres. **(I**-**K)** Chemosensitivities to 5-FU in different cell groups. Student's t test was used for statistic analysis. **(L)** Semi-quantitative RT-PCR analysis of ATP-binding cassette (ABC) transporters. PCR was carried out for 26 cycles. The relative densities of RT-PCR bands are expressed relative to GAPDH (bar graph). Scale bar: 100 μm. Student's t test was used for statistic analysis, ^*^p< 0.05; ^**^ p < 0.01; ^***^ p < 0.005. Y represents Y27632.

To test whether Sox2 is required for maintaining the characteristics of CRC stem-like cells, the spherogenicity of each group of cells was evaluated. The SW620-shRNA control group had the ability to form solid and smooth rounded spheres from a single cell (Figure 4A), while SW620-shRNA Sox2 cells only formed unconsolidated and small irregular cell-masses (Figure 4B). In addition, knocking down Sox2 expression in SW620 spheres resulted in the loss of the spheroid phenotype, the cells flattened on the plate and subsequently differentiated (Figure 4C and 4D).

Y27632 not only reduced spherogenicity of SW620 cells (Figure 4E), but also resulted in the instability of spheres (Figure 4F). However, when Y27632 was withdrawn from the cultures of the SW620-shRNA control, SW620-mock cells or SW620-Sox2 cells, a sub-population of elongated cells became rounded and formed spheres again under serum-free culture conditions (Figure 4G). Y27632 obviously inhibited the activation of phosphorylated filamin A (Supplementary Figure 4), which locates in the downstream of ROCK signal pathway [16], however, Y27632 did not change the expression levels of Sox2 in spheroid cells (Figure 4H).

Since Sox2 expression was always found in the rounded cells, we then analyzed whether rounded cells expressed putative cancer stem cell markers. We immonostained CD133, which is an important surface marker of CRC stem cells [17] in human CRC specimen. We found that CD133 was highly expressed in the rounded and dispersed cells rather than in elongated cells (Supplementary Figure 5).

Previous research demonstrated that Sox2 expression increased chemoresistance in prostate cancer [6], suggesting that down-regulation of Sox2 might be a promising strategy to assist cancer therapy. To test this hypothesis, the cells were treated with 5-fluorouracil (5-FU) to analyze chemosensitivity. SW620-shRNA Sox2 cells were more sensitive to 5-FU, while Sox2-overexpressing cells were more resistant to 5-FU in comparison with each group of control cells (Figure 4I, 4J and 4K). It has been reported that the expression of ABCC1, ABCC3 or ABCC6 was upregulated in multi-drug resistant cancer [18–20]. In the present study, elevated expression of ABCC1, ABCC3 and ABCC6 was observed in SW620-Sox2 spheres (Figure 4L).

## DISCUSSION

The present work reveals that Sox2 is pivotal in the regulation of CRC motility and progression. Sox2 was detected in the early stages of the disease, suggesting that up-regulation of Sox2 might be an early event in colorectal carcinogenesis. The data shows that the expression of Sox2 in tumors graded as Duke's B was higher than that in Duke's C or Duke's D graded tumors. We hypothesize that Sox2 is essential for the invasive capacity of CRC cells, potentially upregulating target genes required to break through the intestinal submucosa. The GSEA assay in the study revealed that the Sox2-targeted gene set was enriched in poorly differentiated tumors, and that Sox2 expression was correlated with tumor differentiation, grade and recurrence in CRC specimens. The results from other research groups also indicated that Sox2 is up-regulated in several types of cancer including colorectal cancer, suggesting it is associated with downstream events of carcinogenesis, including invasion and metastasis [6–8].

Recent studies have revealed extensive genetic and phenotypic variation exists within individual tumors. Intratumor heterogeneity affects tumor biology, responses to anti-cancer drugs and prognosis of patients [21, 22]. In clinical CRC specimens, we observed several subclones within the same CRC specimen. We found for the first time that the vast majority of rounded cells locate in poorly differentiated regions, tumor emboli, InF and META, while elongated cells locate in well differentiated regions and luminal structures. In CRC cell lines, we also found rounded and elongated cells, within the metastatic SW620 whole cell population. Furthermore, in both clinical tissues and in the SW620 cells, rounded cells showed higher expression levels of Sox2 than elongated cells. Silencing Sox2 attenuated colony forming capacity, migration, invasion and tumorigenicity. Overexpression of Sox2 enhanced anchorage-independent growth and tumorigencity *in vitro* and *in vivo*. We hypothesize here that if CRC cells are induced to invade or propagate, then transcription of Sox2 would be promoted and as a consequence cell shape would be remodeled. Alteration of cell shape mirrors dynamic cellular processes, such as cell growth, differentiation, reprogramming and movement. Cell shape and cell movement are largely determined by cytoskeleton [4]. High levels of Rho-ROCK signaling are associated with movement of individual cells in a rounded or amoeboid mode. We found that overexpression of Sox2 increased the expression of RhoA and RhoC, while, knockdown of Sox2 repressed the expression of RhoA and RhoC (Supplementary Figure 6). The data presented here shows that knockdown of Sox2 expression increased the ratio of elongated cells within the whole cell population. We speculated that this was caused by an association between Sox2 and the Rho-ROCK signaling pathway. Inhibition of the ROCK pathway by Y27632 not only increased the ratio of elongated cells, but also decreased CRC cell migration speed and invasion capacity. The effect of Sox2 on tumor cell invasion and migration was also confirmed *ex vivo*. Xenografted tumors burden of Sox2-overexpressing cells showed high microvessel density and tumor emboli located in capillaries or vessel-like structures. These intriguing results suggest that CRC cells have the capacity to break into blood vessel because of their enhanced migration and invasion capacities. The high frequency of tumor emboli found in Sox2-overexpressing tumors indicated that Sox2-overexpressing cells were more aggressive than the cells with low Sox2 expression levels.

Recently, two studies reported that high expression level of Sox2 in head and neck squamous cell carcinomas (HNSCC) had beneficial effect on patient survival [23, 24]. Since HNSCC is from squamous cells, while CRC belongs to adenocarcinoma, we speculate that same gene in different kinds of tumor or under different genetic background might play different roles.

Excluding the clonal evolution model, the CSC model provides an explanation for phenotypic and functional heterogeneity among cancer cells [25, 26]. CSCs are believed to be associated with tumor recurrence, a drug resistant phenotype and poor prognosis [27, 28]. Our previous study confirmed the presence of stem-like cells in CRC, which express high levels of Sox2. Sox2 plays an important role in embryonic stem cells (ES cells) and induced pluripotent stem cells (iPS cells) [29], however, the regulatory function of Sox2 in CRC stem cells remains unclear. In this study, knockdown of Sox2 in SW620 spheres resulted in a reduced capacity of spherogenicity. Notably, we found high expression levels of RhoA and RhoC in spheroid cells, particularly those overexpressing Sox2 (Supplementary Figure 6). Furthermore, blockage of ROCK activation also disrupted spherogenicity and invasion capacity. These results support the notion that Sox2 regulates CSCs characteristics through the Rho-ROCK signaling pathway. Further studies investigating the role of Sox2 and Rho-ROCK signaling pathway in CSCs will be very useful in developing novel therapeutic strategies for CRC.

In the clinic, chemoresistance poses the biggest challenge for maintaining disease free periods and causes recurrence of disease because CRC easily acquire multidrug resistance following successful initial therapy [30]. ABC transporters are important for multidrug resistance and several of them are known to be up-regulated in CRC with chemoresistance [31, 32]. Of note, among the ABC transporters, ABCC3 and ABCC6 are direct transcriptional targets of Sox2. Our data shows upregulation of ABCC1, ABCC3 and ABCC6 in SW620 spheres overexpressing Sox2. Hlavata et al found that ABCC1 is up-regulated in tumors versus normal colon tissues [33] and Schmidt *et al* compared the expression profile of different stages of 5-FU resistance in CRC cells and correlated 33 important genes to 5-FU resistance, including ABCC6 [30]. The data provided here demonstrated that Sox2 silencing and Sox2 overexpression were directly correlated to 5-FU chemosensitivity in CRC cells. We speculate that Sox2 might be one of the *bona fide* operators involved in chemoresistance of CRC.

Based on these observations, we propose a model linking the Sox2-ROCK pathway to CRC cell behavior. Elevated Sox2 expression induces activation of the Rho-ROCK pathway, which in turn changes cell morphology and consequently promotes cell migration/invasion, tumorigenesis and self-renewal capacity. Currently, there are few therapeutic approaches to prevent disease progression, locoregional or distal spread and chemoresistance in CRC. In this regard, our observations offer an important molecular target for diagnostic and therapeutic applications. For example, inhibiting Sox2 expression by short peptide combining with Y27632 might be possible strategy to control CRC progression.

## MATERIALS AND METHODS

### Clinical specimens and statement of ethics

136 tissues were obtained and pathologically graded from patients who underwent surgical resections at the Sun Yat-sen University Cancer Center. Tumor differentiation was characterized according to the WHO classification, while the surgical pathologic stage was analyzed according to the TNM classification system of the International Union against Cancer. Collection of clinical data was performed in accordance with the guidelines of the institutional review board. Before sample collection, informed consent was obtained from the patient. Animal handling and care protocols were approved by the institutional animal care and use committee, Sun Yat-sen University.

### Immunohistochemical and immunofluorescent analysis

Expression of Sox2 or CD31 (primary antibodies diluted 1:100, Cell Signaling Technology, Danvers, Mass) was evaluated using immunohistochemical (IHC) staining. For staining quantification, the stained slides were scored by two investigators, who were unaware of the clinical diagnosis. Five random 200 × microscopic fields were photographed using a standard DP71 Light Microscope (Olympus, Monolith, Japan), and Image-Pro Plus 6.0 (Media Cybernetics Co., American) was used for digital photograph analysis. All digital photographs were taken and measured using the same parameter settings. A staining score was defined by using IPP (Image Pro Plus, Media Cybernetics, USA).

For immunofluoresent analysis, the fixed cells were probed with a primary anti-Sox2 antibody (diluted 1:100), and then incubated with a 1:250 diluted Alexa 488 goat anti-rabbit IgG (Thermo scientific, Waltham, MA, USA). Images were acquired using a Zeiss Imager Z1 fluorescence equipped with an AxioCam MRc5 digital CCD camera (Carl Zeiss Microimaging Inc, Oberkochen, Germany).

Antibodies used in this work are listed in Supplementary Table 1.

### Statistical analysis of cell shape

Semi-quantitative assessment of cell shape was performed according to the methods described previously [4]. Cell shape was graded as “0-3” under a 200×magnification. “0” refers to round cells; “1” refers to ovoid cells; “2” refers to elongated cells and “3” refers to spindled cells. Statistical paired t-tests were used to evaluate the significance of differences of cell shape. To test for inter-observer agreement, 39 samples were randomly selected and scored.

### Cell culture

Colorectal cancer cell lines, SW480, SW620 and HT29, were obtained from the cell bank of the Chinese Academy of Sciences (Shanghai, China). The cells were maintained in DMEM/F12 (DF12, Sigma, St. Louis, MO) containing 10% FBS (Hyclone, Utah, USA) at 37°C with 5% CO_2_. To generate suspended spheres, the SW620 cells were acclimated gradually in DF12 containing 10 μg/ml human insulin, 5 μg/ml human transferrin, 10 μM 2-aminoethanol, 10 nM sodium selenite, and 10 μM mercaptoethanol. All cell lines were authenticated by short tandem repeat (STR) profiling. Where indicated, the cells were treated with 10 mM compound Y27632 (selleck chemicals, Houston, TX, USA) for 24 hr to block ROCK activity.

### Protein preparation and western blot analysis (WB)

For protein preparation from fresh tissues (n=8), the tissues were homogenized with homogenizers and lysed in RIPA lysis buffer (50 mM Tris-HCl pH 7.4, 150 mM NaCl, 1% Triton X-100, 0.25% sodium deoxycholate, 0.1% SDS) containing a protease inhibitor cocktail (Roche, Basel, Switzerland). For protein preparation from the cells, the cells were lysed in RIPA lysis buffer directly. The samples were incubated on ice for 30 min after sonication, and then centrifuged at 10,000×g for 5 min at 4°C. Equal amounts of protein were separated by SDS-PAGE and blotted onto PVDF Immobilon-P membranes (GE Healthcare, New York, NY, USA). Antibodies used in this work are listed in Supplementary Table 1. Target proteins were detected by enhanced chemiluminescence (Thermo scientific) after incubation with secondary antibodies.

### RT-PCR

Total RNA (1 μg) was reverse transcribed to cDNA using a reverse transcription kit as described by the manufacturer's instructions (TOYOBO, Osaka, Japan). Primers used in this work are listed in Supplementary Table 2.

### Cell proliferation assay

Cell proliferation was analyzed using the CCK-8 assay kit (DOJINDO, Shanghai), according to the manufacturer's protocol.

### Colony formation assay

Cells mixed with agar were seeded onto six-well plates (Greiner, Berching, Germany) at 5000 cells/well and cultured for 2 weeks. The cell colonies were fixed with methanol and then stained by Giemsa solution.

### Invasion assay

Briefly, 24-well inserts with 8-μm pores (Millicell, Millipore, MA, USA) were coated with Matrigel (BD bioscience, San Jose, CA, USA). The upper chambers were filled with a suspension of 1 × 10^4^ cells cultured in serum-free DF12, while the lower chambers were filled with DF12 supplemented with 10% FBS. After 48hr-incubation, the filters were fixed and stained with crystal violet.

### Xenograft studies

The cells (1×10^6^ cells of each group) were subcutaneously injected into the flanks of 6-week-old female athymic nude mice (Balb/c nude mice). Tumor sizes were measured in two dimensions with calipers every week. Tumor volumes (cm^3^) were calculated using the following formula: V = 1/2 × length × width^2^.

### Cell transfection

Stable Sox2 expression in CRC cells were achieved using pMXs-hSox2 and control plasmids (Addgene plasmid 17218). MISSON shRNA lentiviral plasmids for Sox2 knockdown or MISSION non-target shRNA control plasmids (Sigma-Aldrich, St. Louis, MO, USA) were transduced into SW620 according to the supplier's manual followed by selection using puromycin (Sigma).

### Statistical analysis

Experiments presented in the figures are representatives of three or more different repetitions. The data are presented as the mean values ± SD. Comparisons between groups were evaluated by a two-tailed student's t test and p < 0.05 was considered statistically significant.

## SUPPLEMENTARY MATERIALS FIGURES AND TABLES


