# Supplementary material for: Sox2 modulates motility and enhances progression of colorectal cancer via the Rho-ROCK signaling pathway
Source: Oncotarget. 2017 Oct 10;8(58):98635–45. doi: 10.18632/oncotarget.21709 (PMC5716756; doi:10.18632/oncotarget.21709)
Supplement: Supplementary file 1 [file oncotarget-08-98635-s001.pdf]

Sox2 modulates motility and enhances progression of colorectal cancer via the Rho-ROCK signaling pathway

SUPPLEMENTARY MATERIALS

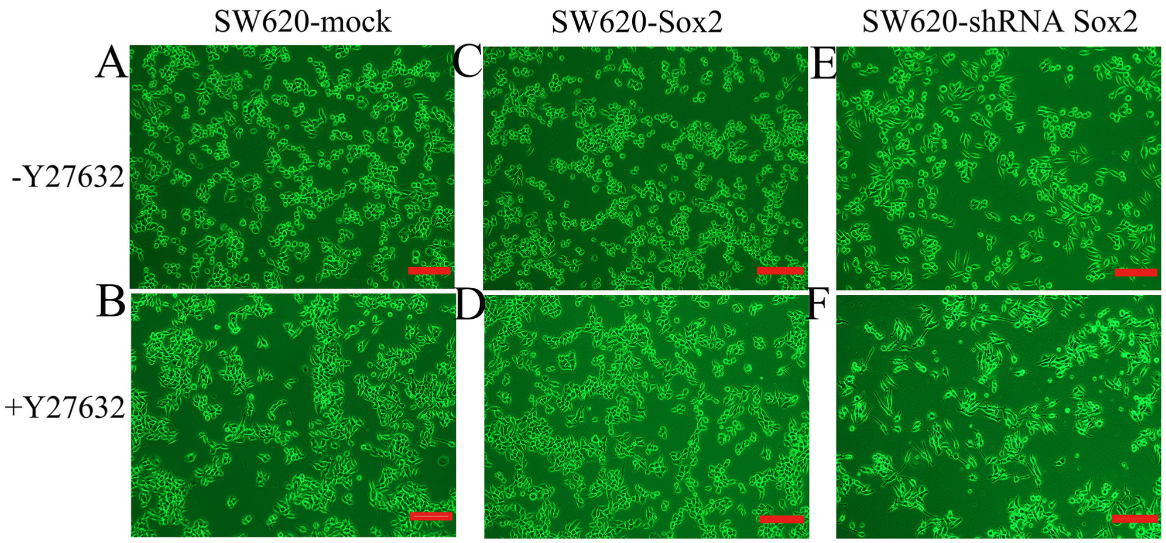

Supplementary Figure 1: Cell morphologies in different cell groups. Y represents Y27632. Scale bar: 100 μm.

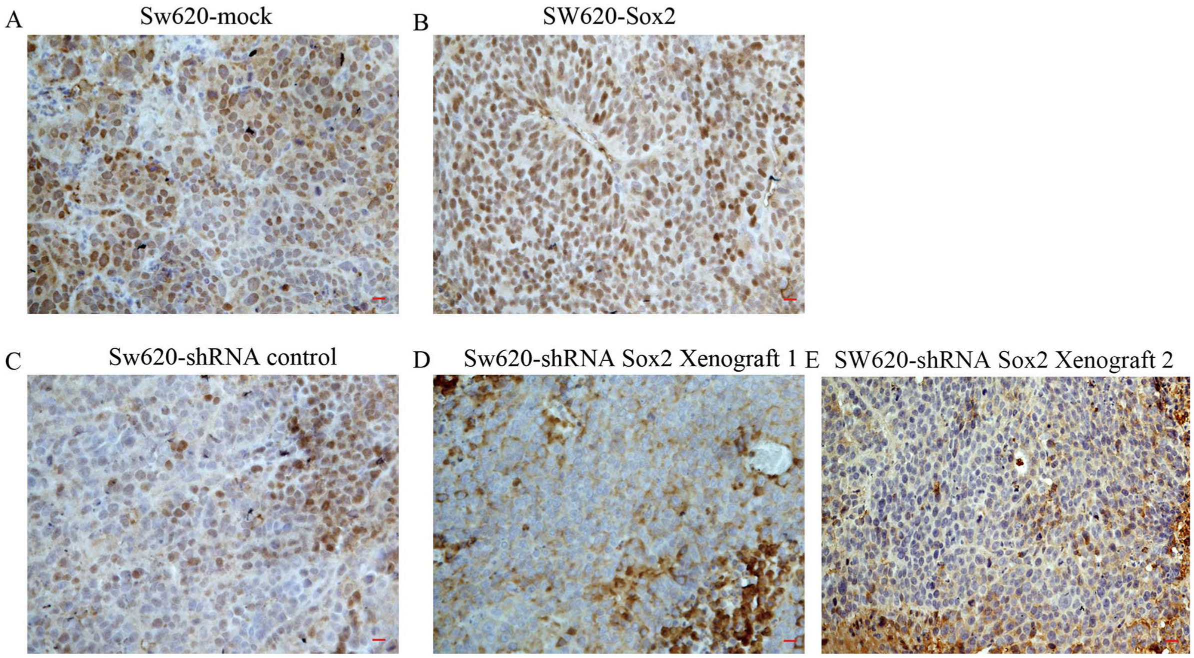

Supplementary Figure 2: Expression of Sox2 in SW620 mock xenograft **(A)**, SW620-Sox2 xenograft **(B)**, SW620 shRNA control xenograft **(C)** and SW620-shRNA Sox2 xenografts **(D and E)**. Representative images are shown, scale bar: 50 μm.

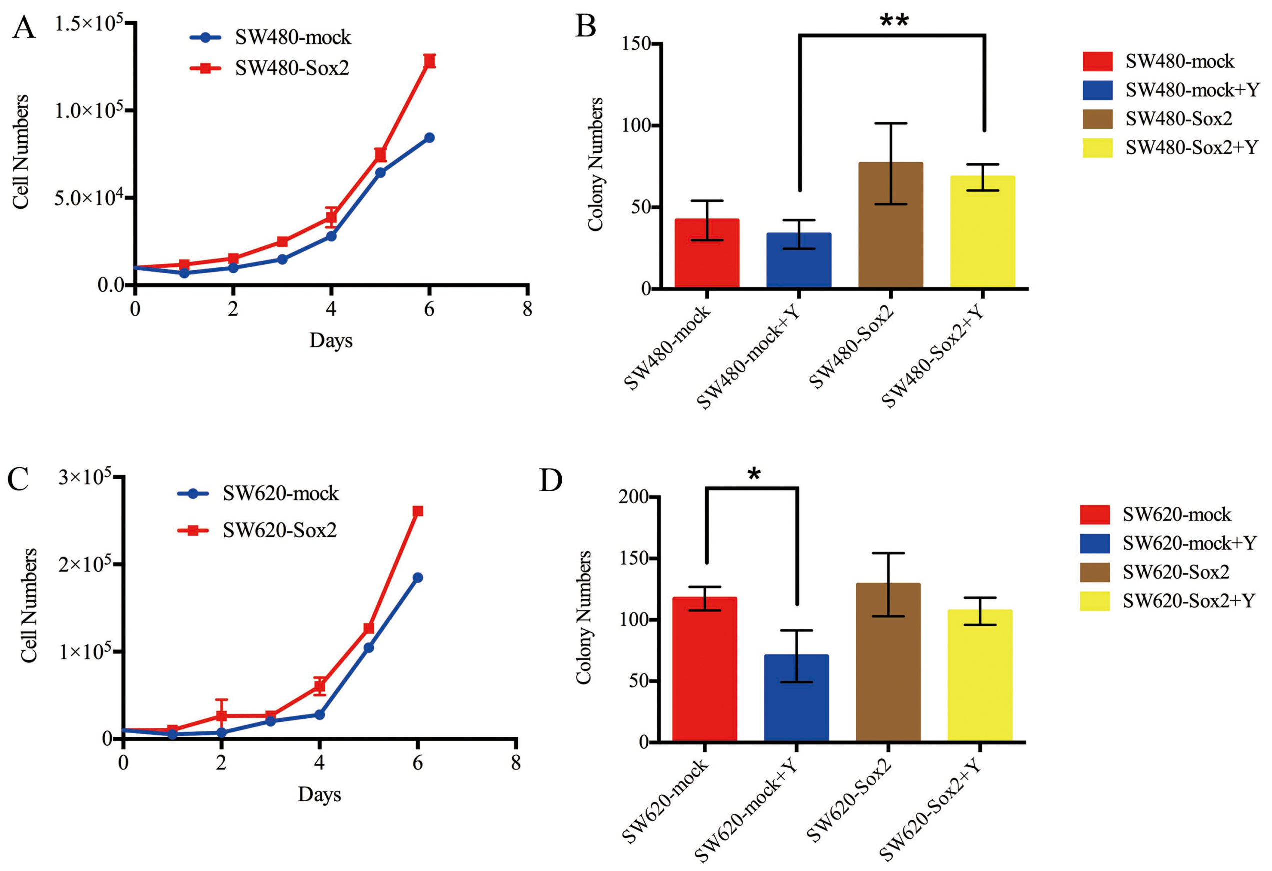

Supplementary Figure 3: (A) Growth assays of SW620-mock cells and SW620-Sox2 cells. **(B)** Quantification of colony numbers of SW620-mock cells and SW620-Sox2 cells. **(C)** Growth assays of SW480-mock cells and SW480-Sox2 cells. **(D)** Quantification of colony numbers of SW480-mock cells and SW480-Sox2 cells. Student's t test was used for statistic analysis, $^{*}p< 0.05$; ** $p < 0.01$; *** $p < 0.005$. Y represents Y27632.

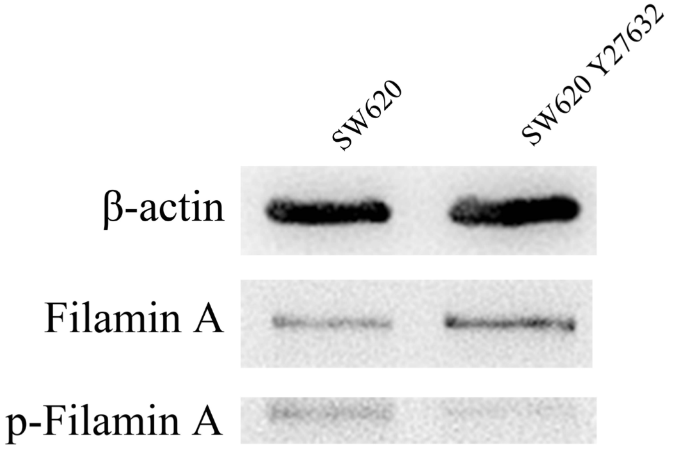

Supplementary Figure 4: WB analysis of filamin A expression. Y27632 inhibited the phosphorylation of filamin A.

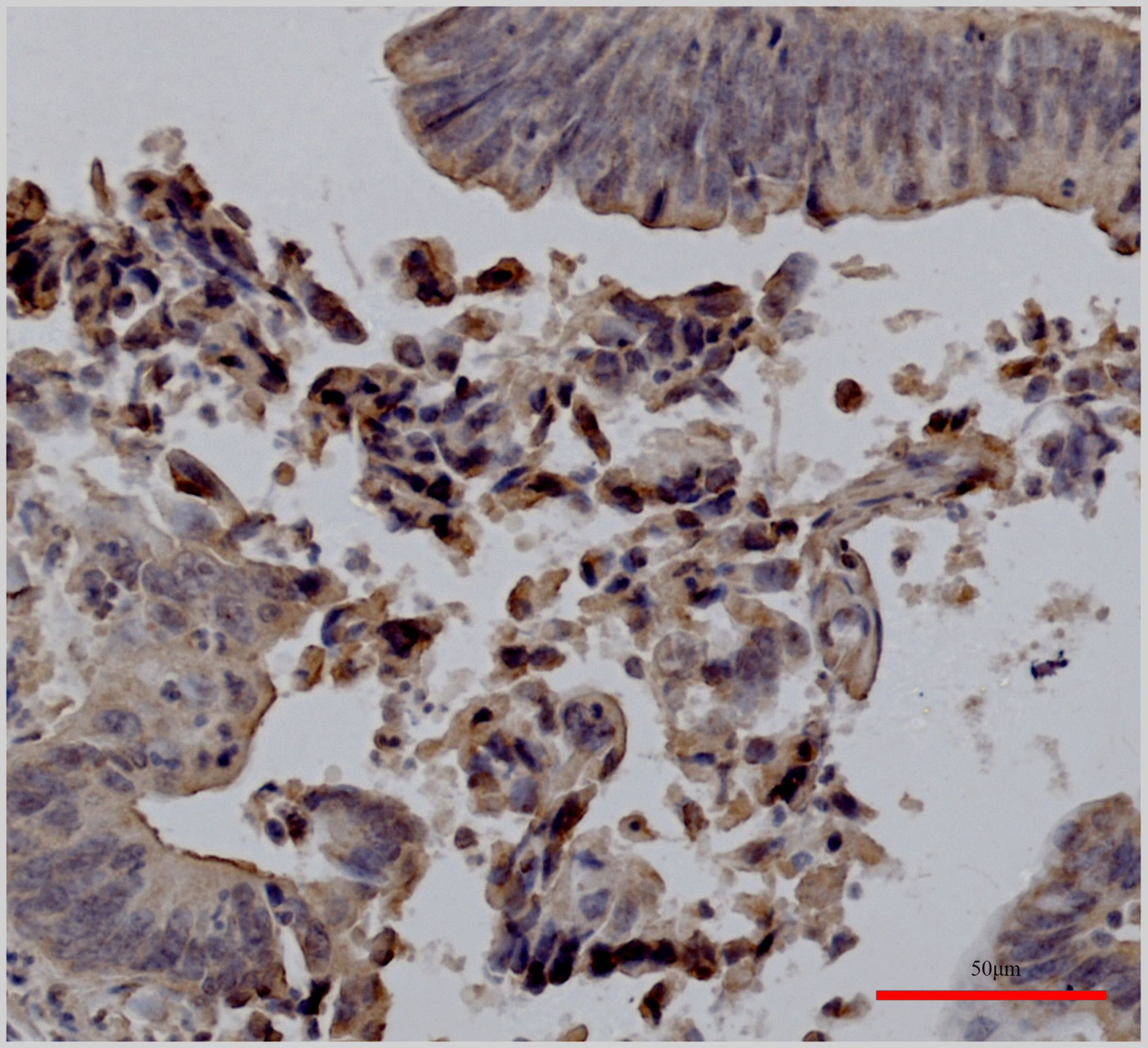

Supplementary Figure 5: Expression of CD133 in human CRC specimen, representative image is shown. Scale bar: 50 µm.

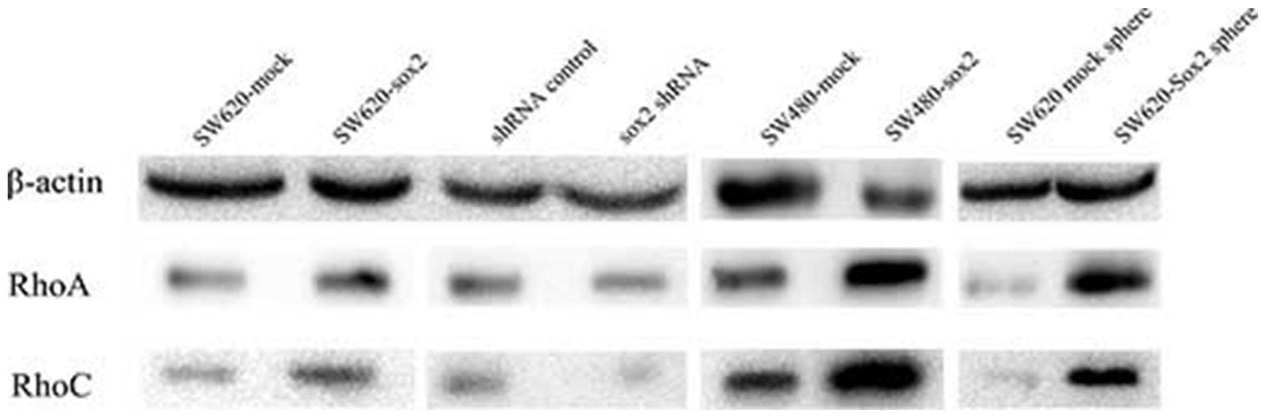

Supplementary Figure 6: WB analysis of RhoA and RhoC. Representative images are shown.

Supplementary Table 1: Antibodies used in this study

Antibody	Dilution Ratio	Producer
anti-Sox2	1:1000 for WB &1:100 for IHC	Cell Signaling Technology
anti-β-Actin	1:1000 for WB	Cell Signaling Technology
anti-RhoC	1:1000 for WB	Cell Signaling Technology
anti-RhoA	1:1000 for WB	Cell Signaling Technology
anti-Rabbit IgG	1:2500 for WB	Cell Signaling Technology
anti-CD31	1:300 for IHC	Cell Signaling Technology
anti-GAPDH	1:250 for WB	Santa Cruz
anti-Filamin A	1:1000 for WB	Cell Signaling Technology

Supplementary Table 2: Primers used in this study

Gene	Sequence	PCR Product Size (bp)
ABCC1-F	CCTGCCCAGTGGGGATCGGA	721
ABCC1-R	GGAAGCCAGCGCGGACACAT	
ABCC3-F	GTCCTGGCTGGAGTCGCTTT	342
ABCC3-R	GGCGTCCAGCACATTGTTTGG	
ABCC6-F	GCGTGGCTGTCGCTCTTTGGA	233
ABCC6-R	GTCGCTCTGGAAGCCCGCTC	
Sox2-F	ATGCACCGCTACGACGTGA	436
Sox2-R	CTTTTGCACCCCTCCCATTT	
